# Investigating Grapevine Red Blotch Virus Infection in *Vitis vinifera* L. cv. Cabernet Sauvignon Grapes: A Multi-Omics Approach

**DOI:** 10.3390/ijms232113248

**Published:** 2022-10-31

**Authors:** Arran C. Rumbaugh, Blythe Durbin-Johnson, Emily Padhi, Larry Lerno, Raul Cauduro Girardello, Monica Britton, Carolyn Slupsky, Mysore R. Sudarshana, Anita Oberholster

**Affiliations:** 1 United States Department of Agriculture, Department of Viticulture and Enology, University of California Davis, One Shields Avenue, Davis, CA 95616, USA; 2 Genome Center, University of California Davis, One Shields Avenue, Davis, CA 95616, USA; 3 Department of Food Science and Technology, University of California Davis, One Shields Avenue, Davis, CA 95616, USA; 4 Department of Viticulture & Enology, University of California Davis, One Shields Avenue, Davis, CA 95616, USA; 5 United States Department of Agriculture, Department of Plant Pathology, University of California Davis, One Shields Avenue, Davis, CA 95616, USA

**Keywords:** grapevine red blotch virus, grape metabolism, grape ripening, virus-induced gene silencing, plant-pathogen interactions

## Abstract

Grapevine red blotch virus (GRBV) is a recently identified virus. Previous research indicates primarily a substantial impact on berry ripening in all varieties studied. The current study analyzed grapes’ primary and secondary metabolism across grapevine genotypes and seasons to reveal both conserved and variable impacts to GRBV infection. *Vitis vinifera* cv. Cabernet Sauvignon (CS) grapevines grafted on two different rootstocks (110R and 420A) were analyzed in 2016 and 2017. Metabolite profiling revealed a considerable impact on amino acid and malate acid levels, volatile aroma compounds derived from the lipoxygenase pathway, and anthocyanins synthesized in the phenylpropanoid pathway. Conserved transcriptional responses to GRBV showed induction of auxin-mediated pathways and photosynthesis with inhibition of transcription and translation processes mainly at harvest. There was an induction of plant-pathogen interactions at pre-veraison, for all genotypes and seasons, except for CS 110R in 2017. Lastly, differential co-expression analysis revealed a transcriptional shift from metabolic synthesis and energy metabolism to transcription and translation processes associated with a virus-induced gene silencing transcript. This plant-derived defense response transcript was only significantly upregulated at veraison for all genotypes and seasons, suggesting a phenological association with disease expression and plant immune responses.

## 1. Introduction

Geminiviruses are responsible for detrimental effects on crop yield and quality worldwide. The international trading of agricultural materials has led to the rapid spread of geminiviruses between continents and the evolution of new virulent strains through recombination and mutation events. There are currently fourteen genera in the *Geminiviridae* family of viruses (*Becurtovirus*, *Begomovirus*, *Capulavirus*, *Citlodavirus*, *Curtovirus*, *Eragrovirus*, *Grablovirus*, *Maldovirus*, *Mastrevirus*, *Mulcrilevirus*, *Opunvirus*, *Topilevirus*, *Topocuvirus*, and *Turncurtovirus*) consisting of 520 different species [1]. All members contain a circular single-stranded (ss) DNA genome, either mono- or bi-partite, with a distinct intergenic region that includes a nonanucleotide motif that functions as the origin of replication [2]. Geminiviruses encode viral proteins via bidirectional transcription in the virion-sense and complimentary-sense.

Like all viruses, geminiviruses must hijack and reprogram the host’s cellular machinery to successfully create an infection. Upon infection, DNA of the geminiviruses require DNA polymerase for replication; therefore, they must enter the nuclei of the host cells where the ssDNA is replicated through dsDNA intermediates [3]. These dsDNA intermediates form dsRNAs that are well documented to be associated with antiviral RNA silencing [4,5]. The dsRNAs are cleaved by Dicer-like (DCL) proteins to form small (~21–24 nt) RNAs (sRNAs) that mediate RNA silencing in plants and are classified into two groups: microRNAs (miRNAs) and short interfering RNAs (siRNAs) [5]. The production of siRNAs leads to several processes, such as degradation of existing RNA (post-transcriptional gene silencing) or targeting DNA for methylation (transcriptional gene silencing) [6]. Tolerant or resistant plant cultivars are known to activate this silencing pathway to lower viral titer levels to achieve an antiviral state [4]. To combat this plant immune response, geminiviruses encode distinct suppressors of RNA silencing leading to abnormalities in plant development which may result in symptoms [7]. Revealing these plant-pathogen interactions remains crucial in understanding host resistance to geminiviruses.

In 2012, the first geminivirus to infect *Vitis vinifera* was identified: Grapevine red blotch virus (GRBV), a species of the *Grablovirus* genus. GRBV is the causative agent for grapevine red blotch disease (GRBD) and has been identified in vineyards across the United States [8,9,10,11,12], as well as several locations internationally [13,14,15,16,17]. Symptoms of GRBD include red blotches on leaves as well as reddening of primary, secondary, and tertiary veins on red varieties and chlorotic regions within leaf blades and marginal burning similar to potassium deficiency in white varieties. Foliar levels of specific amino acids, sugars, phenolics, and terpenoids are reported to be higher in infected grapevines [18]. GRBV substantially impacts berry ripening in all varieties examined so far, causing variable impacts on primary and secondary metabolites, depending on the site and season [19,20,21,22,23]. Most notably is the impact on the phenylpropanoid pathway [22], responsible for flavonoid biosynthesis, which are essential compounds in wine grapes due to their organoleptic properties. The economic impact to vineyards in the United States could reach $68,548/ha with few mitigation strategies available to the industry [24].

Several studies have analyzed how genotypic and environmental factors influence disease outcomes [25,26,27,28] in cassava [29,30], tomatoes [26,31], as well as in grapevines [22,32]. Since grapevine rootstocks can impact grapevine physiology and metabolism, they contribute to plant-pathogen interactions [19,32]. For instance, differences in vigor, resulting in greater shoot length and leaf area, may impact metabolism in the leaves and fruit of grapevines, consequently affecting the final wine composition [33]. In addition, macro and microclimate fluctuations have also been shown to contribute to pathogen-plant interactions [29,34,35] and should be considered.

In this study, we investigated the impact of GRBV infection on grape metabolism during ripening under the influence of genotypic and environmental factors using transcriptomic and metabolomic approaches. This investigation set out to further understand plant-pathogen interactions in GRBV infections and uncovered a phenological association with the expression of a transcript encoding for a DCL protein. Here, we discuss the alteration of transcriptional networks associated with plant-pathogen interactions and a DCL protein in grapevines infected with GRBV.

## 2. Results

### 2.1. Influence of Genotype and Season on Grape Metabolism

**Figure 1:**
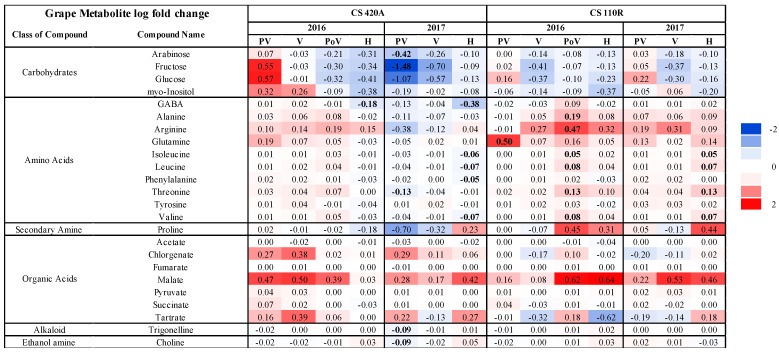
Log fold changes of primary metabolite concentrations through ripening in Cabernet Sauvignon grapes grafted onto 110R and 420A rootstocks in 2016 and 2017. Negative values (blue) indicate a decrease in concentration and positive values (red) indicate an increase in concentration in RB(+) grapes compared to RB(−) grapes. Color gradient indicates the size of log fold change. Values in bold indicate a significant difference (*p* < 0.05, FDR correction). CS = Cabernet Sauvignon, PV = pre-veraison, V = veraison, PoV = post-veraison, and H = harvest.

Grape samples were collected from Cabernet Sauvignon (CS) grapevines that were grafted on two different rootstocks (110R and 420A) and planted in 1993. Fifty grapevines (25 vines each for healthy (RB(−)) and diseased (RB(+)) grapes) in 2016 and 2017 were utilized for metabolomic and transcriptomic analysis. Grapes were collected at four different ripeness stages in 2016 and three different ripeness stages in 2017. Further details of viticultural practices and sampling are discussed in Section 2.1. A total of 78 metabolites (24 volatile secondary metabolites, 30 secondary phenolic metabolites, and 24 primary metabolites) were analyzed. Multi-dimensional scaling indicated that ripeness level primarily explained the variability between samples, followed by season, genotype, and finally disease status (Figure S1). Figure 1 displays the log fold change (FC) in concentration of each primary metabolite between RB(−) and RB(+) grapes.

Generally, GRBV increased amino acids and malate concentrations and decreased carbohydrate levels (Figure 1). Malate concentrations were generally higher in RB(+) grapes at all ripeness levels across seasons and rootstocks. Amino acid concentrations were significantly higher in RB(+) grapes at post-veraison for CS 110R in 2016 and harvest for CS 110R in 2017. Whereas carbohydrate concentrations at harvest were lower and malate concentrations were higher in RB(+) grapes. Proline was higher in RB(+) grapes at harvest except for CS 420A in 2016. To determine the significant seasonal and genotypic influences on grape metabolome, the differential expression analysis analyzed the interactions between season, disease status, and rootstock. Seasonal variation played a larger role in CS 420A for primary metabolite concentrations, where fructose, glucose, arabinose, phenylalanine, threonine, and trigonelline were significantly affected by season at pre-veraison. Between CS 110R and CS 420A several amino acids were significantly affected by the difference in rootstock in 2017, but not in 2016. At pre-veraison in 2017, arabinose, alanine, arginine, phenylalanine, threonine, trigonelline, choline, and chlorogenate were significantly affected by rootstock differences, whereas gamma aminobutyric acid (GABA), leucine, isoleucine, phenylalanine, valine, and threonine were significantly affected at harvest in 2017. Amino acid concentrations were generally lower in CS420 RB(+) grapes than RB(−) at harvest, with the opposite being true for CS 110R. In addition, GRBV significantly lowered arabinose and fructose at pre-veraison for CS 420A in 2017 which was not observed in 2016 or for CS 110R rootstock.

**Figure 2:**
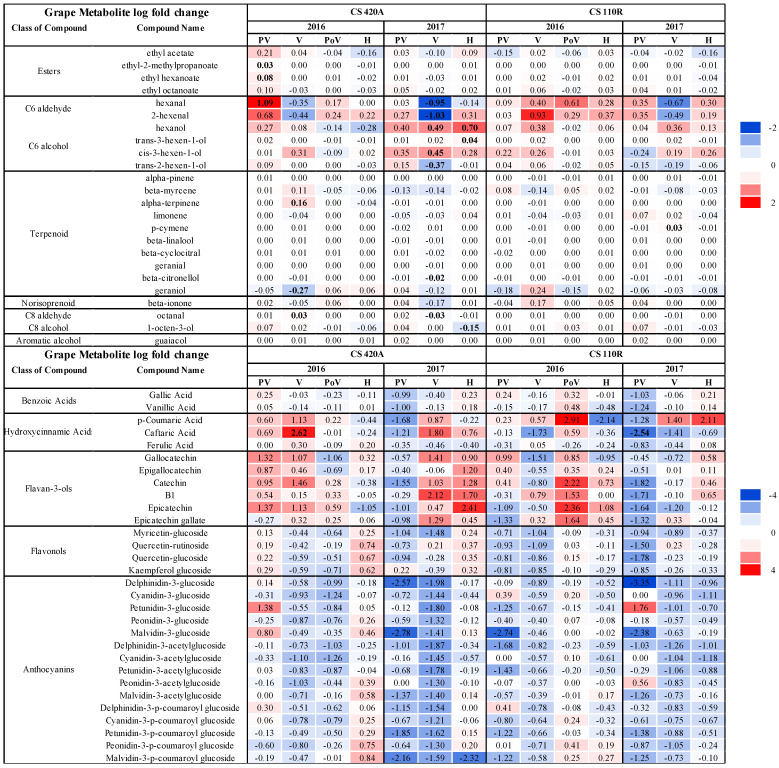
Log fold changes of secondary metabolite concentrations through ripening in Cabernet Sauvignon grapes grafted onto 110R and 420A rootstocks in 2016 and 2017. Negative values (blue) indicate a decrease in concentration and positive values (red) indicate an increase in concentration in RB(+) grapes compared to RB(−) grapes. Color gradient indicates the size of log fold change. Bolded values indicate a significant difference (*p* < 0.05, FDR correction). CS = Cabernet Sauvignon, PV = pre-veraison, V = veraison, PoV = post-veraison, and H = harvest.

Log FC in secondary metabolite concentrations are shown in Figure 2. Few significantly different volatile metabolites were observed, with the most considerable impact occurring in C6 aldehydes, C6 alcohols, and terpenes. Generally, there were increased amounts of C6 aroma compounds at pre-veraison in diseased fruit. Consistent differences were observed between CS 420A and CS 110R in 2017 at veraison, where RB(+) grapes experienced decreases in concentration for C6 aldehydes and increases in the primary C6 alcohols, hexanol, cis-3-hexen-1-ol, and trans-3-hexen-ol, although this was not always significant. Together, our results suggest irregular ripening events in fruit produced on GRBV infected grapevines.

The interaction between disease statues and season indicated that seasonal differences mainly affected 420A rootstock. At veraison, α-terpinene, octanal, and trans-2-hexen-1-ol were significantly affected by season, and hexanol at harvest. Only *p*-cymene was significantly affected by season for CS 110R at veraison. Differences in rootstock significantly affected alpha-terpinene and geraniol at veraison in 2016.

The largest FC differences in phenolic compound concentrations occurred at pre-veraison and veraison, with fewer differences towards harvest. Most notably, there were large decreases in anthocyanin concentrations across season and rootstock as well as transcriptional suppression of the phenylpropanoid pathway at veraison (Figure S2), agreeing with previous results [18,19,20,22,36]. More considerable differences in metabolite concentrations were observed in 2017 than in 2016, with more consistent decreases in anthocyanin concentrations for CS 110R at harvest than CS 420A, agreeing with findings in Martínez-Lüscher et al. [19]. Flavan-3-ol concentrations were mainly higher in RB(+) grapes, yet consistent trends across ripeness level, season, and rootstock were not observed. The interactions between disease status and season or rootstock were not significant for any of the phenolic compounds. However, rootstock differences significantly affected caftaric acid at veraison in both seasons. Caftaric acid was higher for CS 420A in both seasons and lower for CS 110R RB(+) in 2016 grapes at veraison potentially indicating grapevine genotype affects GRBV infection (Figure 2).

### 2.2. GRBV Delays Berry Ripening through Induction of Defense Processes, Photosynthesis, and Auxin Pathways

**Figure 3:**
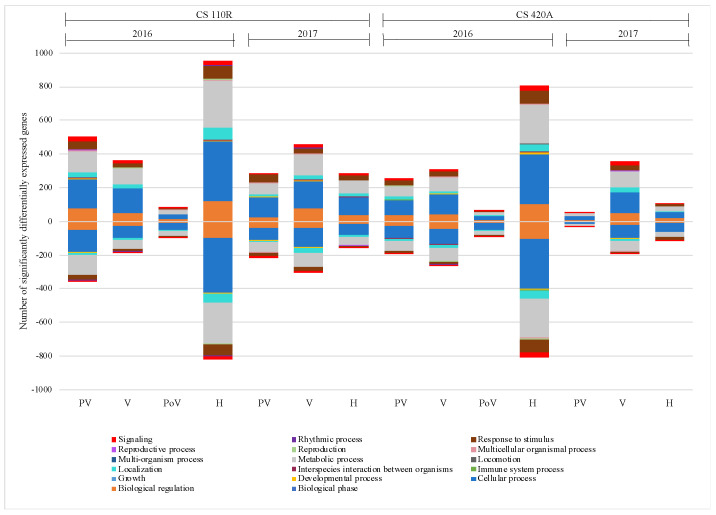
Number of significantly (*p* < 0.01) differentially expressed genes at each ripeness level across genotype and season. Different coloring indicates different gene ontology classifications based on biological processes. Negative values indicate significantly down regulated genes and positive values indicate significantly upregulated genes. CS = Cabernet Sauvignon, PV = pre-veraison, V = veraison, PoV = post-veraison, H = harvest.

RNA-seq was utilized to sequence the transcriptome of the CS grapes. Like the metabolite profiling, the differences in ripeness level predominantly explained the variance in the grape transcriptome, followed by season, rootstock, and then disease status (in descending order of effect, Figure S3). Differential expression (DE) analysis was performed on all trimmed and normalized reads. Significantly (*p* < 0.01) differentially expressed genes (DEGs) for each rootstock, season, and ripeness level are shown in Figure 3. In general, there were fewer DEGs in 2017 than in 2016 for both rootstocks and fewer DEGs for CS 420A than CS 110R, concurrent with primary metabolite results. Gene ontology analysis (GO) determined the main processes impacted were biological regulation, cellular processes, localization, metabolic processes, and response to stimulus.

**Figure 4:**
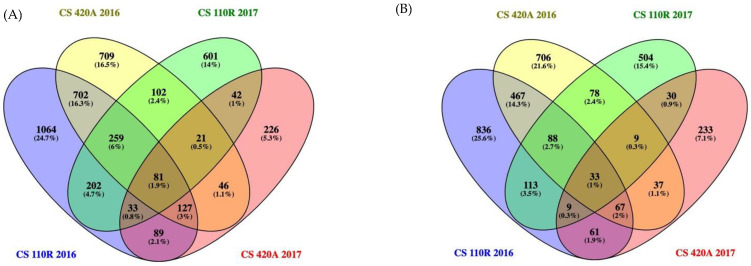
Venn Diagram of upregulated differentially expressed genes (**A**) and downregulated differentially expressed genes (**B**) for each rootstock and season. The genes at each ripeness level were pooled for each rootstock and season combination to find the conserved up and downregulated genes due to GRBV infection. CS = Cabernet Sauvignon.

**Figure 5:**
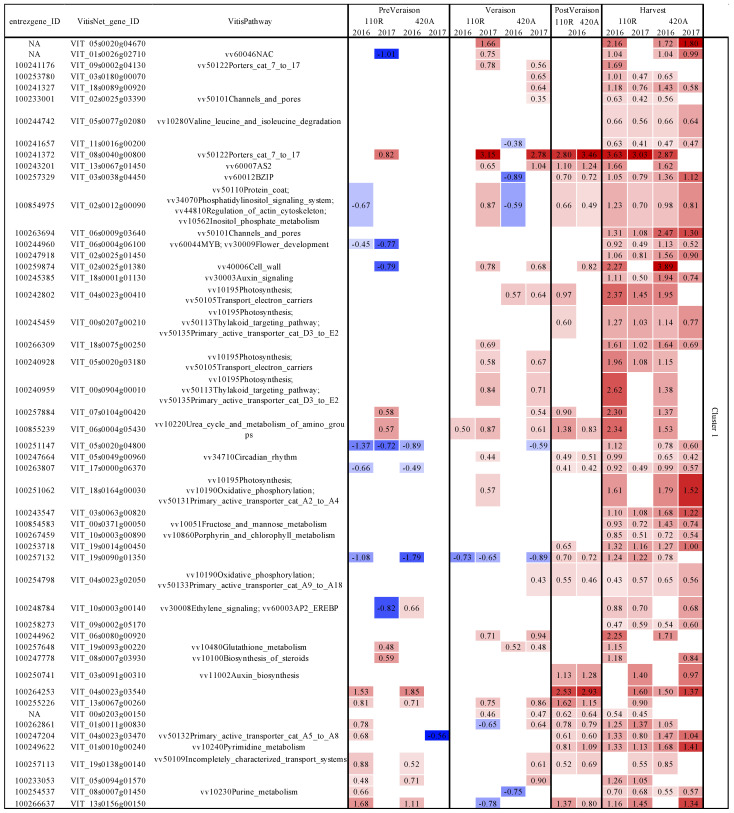

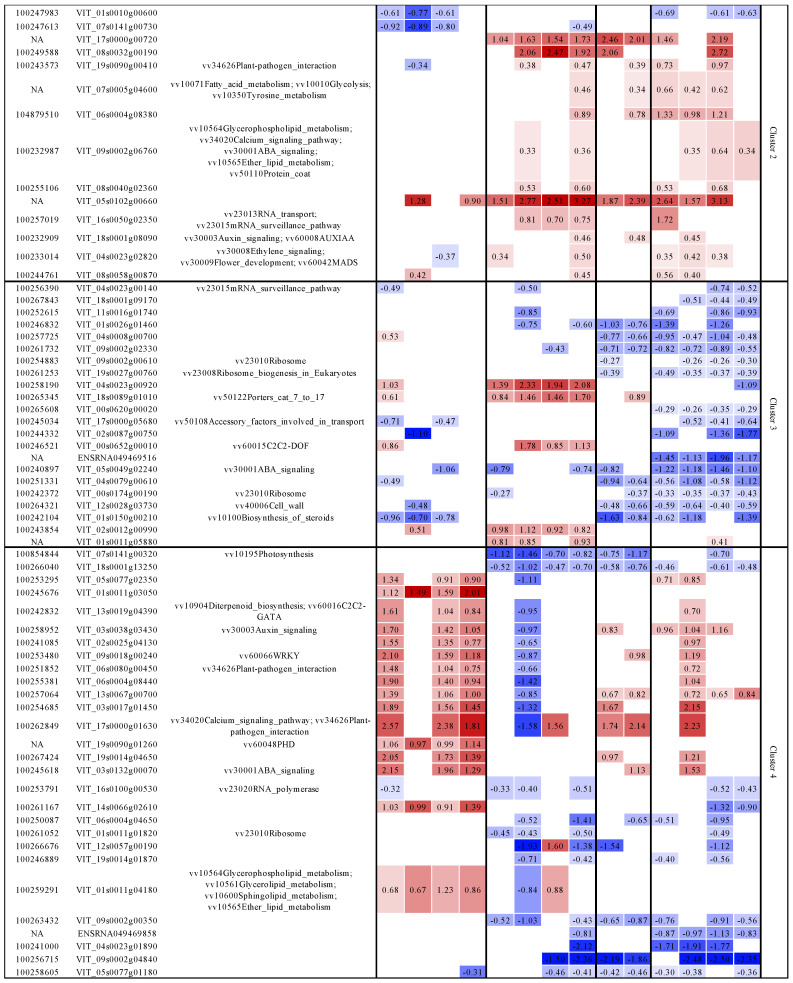
Log fold change of conserved genes affected by GRBV infection based on results from the Venn Diagram in Figure 4. Transcripts are grouped together in clusters based on dendrogram output. Negative values (blue) indicates a decrease in gene expression in diseased grapes and positive values (red) indicate an increase in gene expression in diseased grapes compared to healthy grapes. Color gradient indicates the size of log fold change. 110R = Cabernet Sauvignon on rootstock 110R and 420A = Cabernet Sauvignon on rootstock 420A.

To determine the consistent responses across genotype and season, all the significant DEGs of each rootstock/season were pooled across ripeness levels to generate a Venn Diagram (Figure 4). Figure 4A depicts all the commonly upregulated genes (81), and Figure 4B represents all the commonly downregulated genes (33). A dendrogram separated these 114 genes into four different clusters of 50, 14, 22, and 28 genes. The VitisNet [37] (http://vitis-dormancy.sdstate.org (accessed on 12 January 2021)) and VitisPathway [38] (http://www.rit.edu/VitisPathways (accessed on 12 January 2021)) databases were used for gene annotation of these genes in each cluster, and a heatmap was used to visualize the regulation of these 114 genes due to the viral infection (Figure 5).

Cluster one mainly showed induction of transcripts in GRBV grapes from post-veraison to harvest (Figure 5). Of the 50 genes, seven were associated with energy metabolism (photosynthesis and oxidative phosphorylation), 12 were associated with transportation processes, three were associated with amino acid metabolism, and four with hormone processes. Three of the latter genes were related to auxin-mediated processes. Transcript VIT_08s0040g00800, encoding an auxin-induced protein, was highly upregulated from post-veraison to harvest, and in 2017 from veraison.

Cluster two was moderately induced due to GRBV infection at veraison to harvest. This cluster was associated with lipid metabolism, hormone signaling, and translation processes. GRBV induced one gene related to plant-pathogen interactions, VIT_19s0090g00410, at veraison in 2017 and harvest in 2016 for both rootstocks, potentially indicating that seasonal conditions may relate to the induction of plant responses to viral infection. Some of the largest differences in cluster two were transcripts encoding for currently uncharacterized proteins. The 22 genes in cluster three were mainly suppressed in GRBV infected grapes at harvest. These genes are related to translation, ABA signaling, and cell wall metabolism. One of the genes in this cluster, VIT_04s0023g00920, which is re involved in RNA virus-induced gene silencing (Uniprot; https://www.uniprot.org (accessed on 19 January 2021)) was consistently upregulated at veraison. Lastly, genes in cluster four were mainly induced at pre-veraison due to GRBV infection and associated with plant-pathogen interactions, defense responses (WRKY transcription factor), ABA signaling, and auxin signaling. A few other genes were also suppressed at veraison to harvest, and these were mainly associated with translation processes. Interestingly, only CS 110R grapes in 2017 did not follow this trend. Instead, GRBV infection led to a suppression of the transcripts in the plant-pathogen interaction pathway, WRKY, and auxin signaling at veraison, followed by induction at harvest. The only time that anthocyanin concentrations were significantly impacted at harvest was for CS 110R in 2017, suggesting a differential response to GRBV infection by CS 110R versus CS 420A in 2017, and this was not observed in 2016.

### 2.3. GRBV Induces Genes Involved in Plant-Pathogen Interactions

All the DEGs were also used to construct a weighted gene co-expression network analysis (WGCNA). The results from the WGCNA indicated that the grouping of genes was mainly due to the difference in ripeness levels and the impact of the disease was indistinguishable (Figure S4). Thus, differential co-expression analysis was performed on the DEGs. Differential co-expression analysis aims to identify coordinated expression patterns that differ across conditions. Our study compared differences in gene co-expression between healthy and diseased grapes to determine networks of genes that are impacted due to the virus. Due to the entire network of correlation differences being too large to thoroughly analyze at an adjusted *p* < 0.05 (FDR correction), we used adjusted *p*-values < 5.0 × 10^−6^, which afforded 185 correlations.

Out of these 185 correlations, four contained more than four genes. Three of these networks gained co-expression, and one lost co-expression due to GRBV infection in grapes. One gaining co-expression was related to sugar metabolism, ethylene signaling, cell wall metabolism, and nucleotide sugar metabolism (Figure S5). Another had a centralized gene that was gaining co-expression with several genes (Figure S6). The transcript in the center is a calcium-binding protein (VIT_14s0006g01400) associated with plant-pathogen interactions. The exterior transcripts encoded for transcription factors, WRKY (VIT_17s0000g01280), bHLH (VIT_17s0000g00430), and Zf-HD (VIT_14s0108g00810), glycolysis (VIT_17s0000g03280), tyrosine metabolism (VIT_17s0000g03280), fatty acid metabolism (VIT_17s0000g03280), sucrose metabolism (VIT_12s0057g00700), a SWEET sugar transporter (VIT_1s0000g00830), and auxin transport (VIT_01s0011g04640; Table S1).

**Figure 6:**
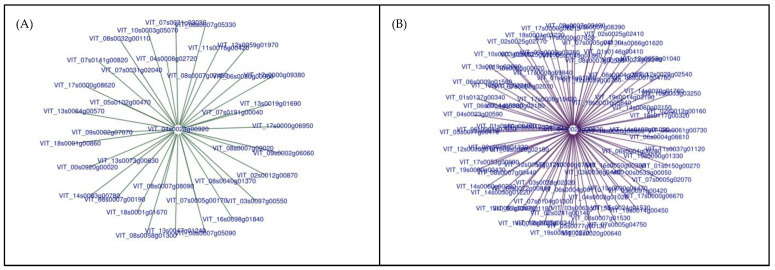
Two networks produced through differential co-expression analysis, one showing a gain in co-expression (**A**) and one showing a loss in co-expression (**B**). The centralized gene is VIT_04s0023g00920, which encodes for a dicer-like protein The transcripts on the exterior are associated with (a) transcription and translation processes or (b) with metabolite synthesis and energy metabolism.

The other two networks that contained more than four genes are shown in Figure 6. The centralized gene gains (Figure 6A) or loses (Figure 6B) co-expression with several encompassing genes. Interestingly, the gene in the center of both figures is the same, VIT_04s0023g00920, and encodes a dicer-like (DCL) protein, specifically DCL2. Our data suggests a transcriptional shift caused by GRBV reallocating the co-expression of this gene in diseased grapes. Figure 6A demonstrates that this gene gains co-expression with genes responsible for flower development, translation, and transcription processes in GRBV fruit (Table S2). Simultaneously, there is a loss of co-expression (Figure 6B) with genes associated with plant-pathogen interactions, flavonoid biosynthesis, amino acid metabolism, carbohydrate metabolism, transport, cell wall metabolism, and oxidative phosphorylation (Table S3). The one gene associated with plant-pathogen interactions is again a calcium-binding protein (VIT_02s0241g00140). The transcript in the flavonoid biosynthesis pathway encodes for the chalcone-flavanone isomerase family of proteins (VIT_13s0067g02870), which precedes the synthesis of flavonols, flavan-3-ols, anthocyanins, and proanthocyanidins in the phenylpropanoid pathway.

**Figure 7:**
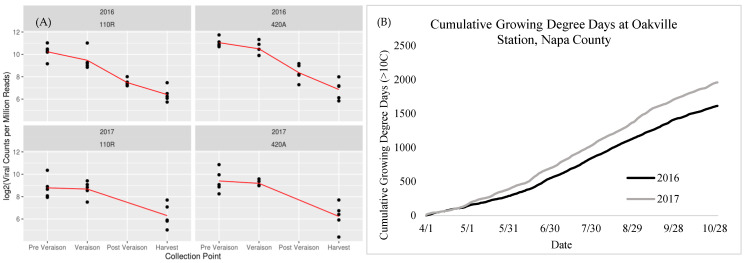
Comparison of viral gene expression in each season (**A**) and cumulative growing degree days in each season (**B**). 110R = Cabernet Sauvignon on 110R rootstock and 420A = Cabernet Sauvignon on 420A rootstock. Viral counts consist of the two mRNAs (sense strand and antisense strands) thought to translate the five proteins encoded by the GRBV genome.

Analyzing the DE of DCL2 revealed a significant (FDR adjusted *p*-value < 0.05) induction only at veraison for both seasons and rootstocks (Table 1). More considerable differences in DE were observed between 2016 than 2017. These data were compared to the viral gene expression, which was determined by overlaying the GRBV genome with the grape RNA-seq data. The six open reading frames of the GRBV genome encode for five proteins, and these five proteins are thought to be translated from two mRNAs: the sense strand and the antisense strand. Therefore, the counts from open reading frames 1, 2, and 3 were combined (sense strand), and 4, 5, and 6 were combined (antisense strand) to determine viral gene expression in the diseased grapes (Figure 7A). Expression of viral genes was highest at all points at pre-veraison, with a slight decrease at veraison and more drastic decreases until harvest.

## 3. Discussion

GRBV is known to inhibit ripening processes in grapes leading to decreases in carbohydrate levels, increases in malic acid, and variable impacts on secondary metabolites depending on seasonal and genotypic factors [19,20,21,22,23,39]. In the current study, GRBV suppressed the phenylpropanoid pathway at veraison resulting in decreased anthocyanin concentrations through ripening. Phenylalanine concentrations, the amino acid that initiates the phenylpropanoid pathway and the synthesis of flavonoids, was generally higher at pre-veraison and lower by harvest in diseased fruit compared to healthy fruit. Although anthocyanins were affected through ripening, the decreases in phenylalanine accompanied with fewer anthocyanins being lower by harvest potentially indicates a delayed biosynthesis of anthocyanins in GRBV infected grapes, which was generally recovered by harvest. Our study corroborated previous results indicating that GRBV inhibits ripening events in grapes resulting in lower carbohydrate and anthocyanin concentrations, with higher malic acid, amino acid, and C6 aroma compound concentrations [19,20,22,39].

In the current study, larger differences in secondary metabolites concentrations due to GRBV infection were seen in 2017 than in 2016. This was also observed in Rumbaugh et al. [39], where it was hypothesized that the higher temperatures in 2017 potentially increased plant defense responses, leading to fewer differences in primary metabolite concentrations. However, simultaneously it acted as a secondary stressor for RB(+) fruit in terms of secondary metabolites, such as anthocyanins, which are more sensitive to elevated temperatures, leading to larger differences [40].

GRBV infection was more impacted by seasonal differences in rootstock 420A than 110R, mainly impacting amino acids and carbohydrates suggesting that the pathogenicity of GRBV in 420A is correlated to environmental factors. Differences in rootstock mainly impacted amino acid concentrations in 2017, not in 2016 potentially suggesting that genotypic and environmental differences affect how the grapevine host will interact with GRBV. Together, our results conclude that the genotype 110R, a more drought-tolerant and vigorous rootstock than 420A, has a differential response to GRBV infection over 420A. CS 110R in 2017 did not undergo the same conserved transcriptional response to GRBV as the other rootstock/season combinations (Figure 5), which may have contributed to the lower anthocyanin accumulation at harvest (Figure 2). In addition, there were fewer significant DE genes for 420A than 110R in both seasons (Figure 3). Rootstock susceptibility to viral infection is an ongoing research topic [19,32,39,41,42,43], with variable conclusions. One study determined that grapevine leafroll-associated virus-3 caused greater impacts on the rootstock 110R compared to 196.17C when grafted onto Albariño grapevines [43], which is similar to the current study. On the other hand, Vondras et al. [32] found that Kober 5BB (*V. berlandieri* × *V. riparia*, similar to 420A) was more impacted than MGT 101- 14 during the infection of multiple grapevine leafroll-associated viruses.

GRBV infection was recently reported to generally decrease volatile aroma compound accumulation in grapes, except for C6 aldehydes and alcohols [39]. C6 aroma compounds are synthesized in the lipoxygenase pathway and participate in plant defense responses and growth and development [44,45]. In healthy grapes, C6 aldehydes typically increase in concentration after veraison with a decrease at harvest due to increased ADH activity. Consequently, ADH converting C6 aldehydes into C6 alcohols consistently increases hexanol levels until harvest and, to a lesser extent, trans-3-hexen-ol [46]. In general, transcripts encoding for LOX enzymes are upregulated before veraison and then suppressed during grape ripening [47]. We observed a premature decrease in C6 aldehydes resulting in an increase in the primary C6 alcohols in diseased grapes in 2017. In addition, there was irregular induction of a LOX transcript due to GRBV infection in 2017, potentially resulting in higher levels of C6 alcohols at veraison and harvest.

GRBV infection decreased levels of carbohydrates and anthocyanins while increasing the levels of malic acid in grapes at harvest, similar to previous findings [19,20,23,36,48]. Blanco-Ulate et al. [22] demonstrated that GRBV causes an impairment to ripening events, mainly affecting the phenylpropanoid pathway in which 68% of the genes were suppressed with concurrent decreases in anthocyanin concentrations. GRBV also induced auxin metabolism while decreasing levels of abscisic acid (ABA) and gibberellin. Auxin is involved in many grape processes, such as cell division and expansion in early fruit development and repressing fruit ripening. One of these processes is malic acid catabolism [49]. In healthy grapes, before veraison, malate is synthesized inside the grape berry through several pathways such as glycolysis and photosynthesis, where it is then stored in the vacuole. At veraison, sugars are no longer utilized for energy metabolism and begin to accumulate in the vacuole. In addition, photosynthetic processes drastically decrease. Thus, to accommodate the energy needs of the berry, there is a switch from carbohydrate utilization to malic acid catabolism [50]. Malate is released from the vacuole and becomes available for energy metabolism (through the TCA cycle and oxidative phosphorylation), amino acid interconversions, and secondary metabolite synthesis, such as flavonoids. In the current study, the consistently elevated levels of malate (Figure 1) and the induction of auxin signaling and photosynthesis from post-veraison to harvest (Figure 5) potentially suggest an imbalance in energy utilization after the onset of veraison. This ultimately could have resulted in the desynchronization of ripening events in GRBV fruit that Martínez-Lüscher et al. [19] theorized.

Differential co-expression analysis in the current work indicated a potential signaling control of a SWEET sugar transporter by a calcium-binding protein (Figure S6). SWEET transporters are known to be associated with plant-pathogen interactions, although it has been challenging to define a clear role due to variability in their responses to pathogen infection [51]. The calcium-binding protein was also co-expressed with bHLH, WRKY, and Zf-HD transcription factors during GRBV infection. Basic helix-loop-helix (bHLH) proteins are a superfamily of transcription factors that have been previously associated in defense responses to tomato yellow leaf curl virus, a geminivirus [52]. Both WRKY and zinc finger homeodomain (Zf-HD) proteins have also been correlated to plant defense-signaling pathways during pathogen infections [53,54]. In addition, signaling crosstalk between auxin and Ca^2+^ has been suggested [55]. Although, Ca^2+^ signals calcium-binding proteins to prompt a physiological response to survive an infection [56], viruses are also adept at utilizing Ca^2+^ for their benefit [57]. The current work suggests that GRBV triggers an association of the calcium-binding protein with auxin transport (a hormone primarily responsible for inhibiting ripening events), carbohydrate metabolism, sugar transport, and transcription factors involved in the plant immune system. Martínez-Lüscher et al. [19] proposed that GRBV likely causes an impairment to carbon translocation mechanisms from source-to-sink, which our research indicates may be controlled by a calcium-binding protein mediating a defense response. Further research is needed to determine the precise role of calcium and calcium-binding proteins during GRBV infection.

Among the plant-pathogen interactions, RNA silencing has been widely documented in geminivirus infections [5,58,59,60]. RNA silencing is a plant response that regulates viral gene expression to fight the infection, mediated by sRNAs. Dicer-like (DCL) proteins are essential enzymes in this response as they produce sRNAs [5]. Most plants encode four DCL proteins (DCL 1–4), where DCL 1 encodes for miRNAs, and DCL 2–4 encodes for siRNAs [61]. Specifically, DCL 2 triggers intercellular silencing in cells adjacent to the initial virus-infected cell [60]. Previous literature has successfully correlated higher levels of siRNA accumulation to symptom recovery and decreases in viral titer levels [58,62].

In the current study, the antiviral transcriptional control observed with DCL2 potentially explains the irregular ripening events observed in the fruit of GRBV-infected grapevines for the past 10 years. For the first time, we revealed a transcriptional shift of DCL2, indicating a loss of allocation of resources for primary and secondary metabolism and energy metabolism. The loss of co-expression with the oxidative phosphorylation pathway may also explain the slight downregulation of this pathway and potentially the increase in malate concentrations previously discussed. The gaining of co-expression of this gene with transcriptional and translational processes further supports that GRBV-infected grapes are potentially favoring RNA silencing as a defense mechanism over normal ripening processes. However, to confirm the present findings, analysis of siRNAs in GRBV-infected grapes is needed which was not able to be performed in this study.

Analyzing the DE of DCL 2 reveals that GRBV infection led to significant induction at veraison in both seasons, which was moderate at post-veraison in 2016, and suppression at harvest in both seasons. A similar study investigating transcriptional responses to grapevine leafroll-associated virus-3 (GLRaV-3) infection observed an analogous induction of DCL 2 at only veraison [63]. Interestingly, research indicates that the onset of foliar symptoms for GLRaVs and GRBV begins at veraison [12,18,64]. After veraison, viral gene expression levels decreased (Figure 7B), which may relate to RNA silencing modifying viral RNAs [65]. Taken together, our data suggests a phenological association with plant immune responses, potentially resulting in the onset of foliar symptoms.

In Chellappan et al. [29], similar work was performed on a geminivirus infecting cassava plants. In this study, fluctuations in temperature regulated the expression of RNA silencing where increases in temperatures increased expression and decreased viral titer and symptoms [29,35]. During our study, cumulative growing degree days were higher in 2017 than the previous year (Figure 6A), with nine days exceeding 35 °C and four days exceeding 40 °C. Consistently in this study, the impact of GRBV on the grape transcriptome was lower in 2017 than in 2016 concurrently with generally lower viral gene expression and higher expression of the DCL 2 transcript. Although viral titer levels were not measured in this study, our results agree with previous studies that temperature affects disease expression [29,35].

## 4. Materials and Methods

### 4.1. Chemicals and Reagents

Sodium acetate, PVP-40, NaCl, sodium citrate, ascorbic acid, 2-undecanone, methanol, chloroform, formic acid, decyl-β-glucopyranoside, acetonitrile, potassium phosphate, HCl, NaOH, were all purchased from Sigma Aldrich (St. Louis, MO, USA). EDTA was purchased from Fisher Scientific (Pittsburgh, PA, USA). Guanidine thiocyanate was purchased from Spectrum Chemicals (New Brunswick, NJ, USA). D_2_O was purchased from Cambridge Isotope Laboratories, Inc. (Tewksbury, MA, USA). 3-(trimethylsilyl)-1-propanesulfonic acid-d6 (DSS-d6) was purchased from Chenomx (Edmonton, AB, Canada)

### 4.2. Plant Material and Sample Collection

Cabernet Sauvignon grapevines (clone 8, Foundation Plant Services, University of California Davis) grafted onto 110R (*V. berlandieri* × *V. rupestris*), and 420A (*V. berlandieri* × *V. riparia*) rootstocks were used for this study. Since rootstocks can impact grapevine physiology and berry composition, they can also potentially impact disease severity. Therefore, rootstocks 110R and 420A were chosen due to their differences in vigor and drought tolerance [39,66]. These grapevines were planted in 1999 at the Oakville Experimental Vineyard (Napa County, CA, USA). The grapevines were trained to a bilateral cordon in a vertical shoot positioned system. Viticultural practices are reported in Rumbaugh et al. [39] and Martínez-Lüscher et al. [19]. From this vineyard block, 60 vines were tested for the presence or absence of GRBV, as well as GLRaV-1 to 4, and strains of 4. For this study, only healthy vines (i.e., vines that tested negative for viruses and did not show symptoms of viral disease, RB(−)) and vines which only tested positive for GRBV, and which are symptomatic (RB(+)) were used as data vines. For each treatment, 20 and 25 data vines were identified in 2016 and 2017, respectively. Data vines were randomly subdivided into five biological replicates of four and five vines, using a random sequence generator for 2016 and 2017, respectively (http://www.random.org.sequences (accessed on 23 May 2016)). Five berries were collected from each data vine randomly (top, middle, and bottom of grape bunches on the outer and inner side of the canopy) for a total of 20 berries per biological replicate. Grapes were sampled four times during ripening at pre-veraison, 50% veraison (berry softening and color change), post-veraison, and harvest for 2016. For 2017, grapes were collected at all the previous points, except for post-veraison due to a heat spike and unexpected rapid increases in sugar content. Grapes sampled were immediately processed upon arrival at the laboratory, and berries were deseeded, frozen in liquid nitrogen, and stored at −80 °C until further analysis.

### 4.3. Total RNA Isolation

Total RNA from each biological replicate across seasons, rootstocks, and collection points was extracted and isolated. Approximately 2 g of fresh weight grape material was mixed with a lysate buffer consisting of 4 M guanidine thiocyanate, 0.2 M sodium acetate, 26 mM EDTA, and 2.6% (*w*/*v*) PVP-40. The samples were then homogenized using a table mill, and then total RNA was isolated using the Qiagen RNeasy Plant Mini Kit in conjunction with the Qiagen PowerClean Pro Cleanup kit (Qiagen, Hilden, Germany). DNA was removed using the Zymo Research RNA Clean & Concentrator-5 Kit (Zymo Research, Irvine, CA, USA). RNA integrity and purity were analyzed using a 2100 Bioanalyzer (Agilent, Santa Clara, CA, USA) and NanoDrop 2000c spectrophotometer (Thermo Fisher Scientific, Waltham, MA, USA), respectively.

### 4.4. mRNA Sequencing and Analysis

Gene expression profiling was carried out using a 3′ Tag-RNA-Seq protocol. Barcoded sequencing libraries were prepared using the QuantSeq FWD kit (Lexogen, Vienna, Austria) for multiplexed sequencing according to manufacturer recommendations. Micro-capillary gel electrophoresis was used to verify the fragment size distribution of the libraries on a Bioanalyzer 2100 (Agilent, Santa Clara, CA, USA). The libraries were quantified by fluorometry on a Qubit fluorometer (LifeTechnologies, Carlsbad, CA, USA) and pooled in equimolar ratios. Up to forty-eight libraries per lane were sequenced on a HiSeq 4000 sequencer (Illumina, San Diego, CA, USA). The sequencing was carried out by the DNA Technologies and Expression Analysis Core at the UC Davis Genome Center, supported by NIH Shared Instrumentation Grant 1S10OD010786-01.

### 4.5. Metabolite Extraction and Quantitation

#### 4.5.1. Volatile Compound Analysis

The berries were finely ground in liquid nitrogen for each biological replicate using an IKA analytical mill (Wilmington, NC, USA). Approximately 0.5 g of freshly ground grape powder was added to a 10 mL amber headspace vial (Agilent Technologies, Santa Clara, CA, USA) containing 1 g of NaCl, 1 mL of 1 M sodium citrate buffer, and 25 µL of ascorbic acid solution. Each vial was spiked with 25 µL of a 0.5 mg/L 2-undecanone internal standard solution. For a QC sample, grapes from all collection points, seasons, and rootstocks were homogenized together in liquid nitrogen and treated as a sample. A QC sample was extracted with each extraction batch and analyzed similarly to determine day-to-day instrumental drift.

Headspace solid-phase microextraction gas chromatography coupled to a mass spectrometer (HS-SPME-GC-MS) was used to analyze the volatile profiles of grape extracts, as in Rumbaugh et al. [39]. Ions were monitored using synchronous scan and selected ion monitoring (SIM). All compounds identified in this study were identified using the SIM mode described in Hendrickson et al. [67]. Samples were analyzed using Mass Hunter software version B.07.00 (Agilent Technologies, Santa Clara, CA, USA). Compounds were semi-quantitatively analyzed using relative peak areas by normalization with 2-undecanone as well as the berry mass. The five biological replicates across all variables were analyzed in triplicate. Compounds were identified by retention time and confirmation of mass spectra ion peaks using the National Institute of Standards and Technology database (NIST) (https://www.nist.gov (accessed on 4 September 2019)). A list of 50 volatile compounds was generated from previous literature and was used for compound identification. A final list of 38 compounds was identified in the grape samples and used for quantitation.

#### 4.5.2. Phenolic Compound Analysis

The homogenized frozen grape powder was analyzed by mixing 1 g of grape material with 4 mL of extraction buffer that consisted of methanol:water:chloroform in a 3:1:1 and 0.1% (*v*/*v*) formic acid. Decyl-β-glucopyranoside was used as an internal standard, and each sample was spiked with 80 μL of 100 mg/L solution (final concentration of 500 μg/L). The sample was vortexed for 30 s, sonicated for 10 min at 4 °C, and then centrifuged at 3220× *g* at 4 °C for 10 min at 4 °C. The supernatant was collected, and 1 mL was diluted to 4 mL using 18 MΩ water. The rest of the supernatant was saved for primary metabolite analysis. The sample was mixed and centrifuged for 10 min at 15,000 rpm. One mL of the diluted sample was transferred to a 2 mL amber vial with a screw cap for analysis. QC samples, which consisted of the same grape material as described in Section 4.5.1, were prepared daily in the same manner for phenolic analysis.

Analyses were carried out on an Agilent 1290 Infinity Ultra-high 150 Performance Liquid Chromatography (UHPLC) system coupled with an Agilent 6545 151 quadrupole time-of-flight (Q-TOF) LC/MS (Agilent Technologies, Santa Clara, CA, USA). The temperature-controlled autosampler was kept at 4 °C. Chromatographic separation was carried out on an Agilent analytical column (2.1 × 150 mm, particle size 2.7 μm) after 2 μL of the sample was injected. Mobile phase A was LC grade water with 0.1% (*v*/*v*) formic acid, and phase B was LC grade acetonitrile with 0.1% (*v*/*v*) formic acid. The chromatographic method was 98% phase A (0–1 min), a gradual decrease from 98% to 20% phase A (1–16 min), a decrease to 2% phase A (16–18 min), which was maintained for 2 min (18–20 min), and finally a linear increase from 2% to 98% phase A over one minute (20–21 min) which was then held for another four minutes (21–25 min). The iso pump and binary pump were set to a flow rate of 0.2 mL a minute. The mass range of the detector was 100–1000 *m/z*, and the rate of detection was set to 2 spectra per second with a cycle time of 1 min. The sheath gas and drying gas temperatures were at 375 °C and 200 °C, respectively. The capillary voltage and nozzle voltage were set to 3500 V and 1000 V, respectively. The nebulizer was set to 50 psi, and the fragmentor voltage set to 100 V. The internal standard eluted at 13.45 min with the mass of 321.2272 *m/z*, 343.2091 *m/z*, and 359.183 *m/z* for H+, Na+, and K+ ionized forms of the internal standard, respectively. The area of the peak of mass 343.2091 *m/z* was used for the normalization of all other compounds identified. A list of 51 phenolic compounds that were previously cited in the literature was utilized for compound identification. The final number of identified compounds in all samples across environmental, developmental, and genotypic factors was 36 compounds which consisted of benzoic acids, hydroxycinnamic acids, flavonoids, and stilbenes.

#### 4.5.3. Primary Metabolite Analysis

For the analysis of primary metabolites, 1 mL of the supernatant from the phenolic hydroalcoholic extraction was utilized. The sample was dried under vacuum for 4 h at 35 °C, suspended in 1 mL of D_2_O, and then dried again under vacuum again for 4 h at 35 °C to reduce the methanol signal [68]. The dried samples were then reconstituted with 1 mL of 10 mM potassium phosphate buffer (pH 6.8), vortexed until completely homogenized, and centrifuged at 14,000× *g* at 4 °C for 5 min. Into a new microcentrifuge tube, 585 μL of the sample was mixed with 65 μL of 5 mM DSS-d6 as an internal standard. Each sample was adjusted to a pH of 6.8 using 1 N HCl or NaOH, and 600 μL was transferred to a 5 mm NMR tube. Samples were stored at 4 °C for no longer than 24 h until the NMR spectra were acquired. Sample acquisition and analysis were performed as in Chin et al. [69,70]. Briefly, the ^1^H NMR spectra of the aqueous samples were acquired at 298 K on a Bruker 600 MHz NMR spectrometer (Bruker BioSpin AG, Fällanden, Switzerland) equipped with a TCI cryoprobe and a SampleJet using the noesypr1d pulse programs. Each spectrum was acquired in approximately 10 min. Chenomx Inc. NMR suite Processor version 8.3 (Edmonton, AB, Canada) was used to identify and quantify primary metabolites in grape. A total of 26 metabolites were identified and quantified, ranging from amino acids, organic acid esters, and carbohydrates.

### 4.6. Statistical Analysis

All metabolites were subjected to differential expression analysis using limma-voom. Log fold changes based on averages and *p*-values were calculated using R (version 4.0.1). All significant metabolite differences were determined by adjusting the *p*-value using a false discovery rate (FDR) test (*p* < 0.05, FDR correction).

Raw reads were processed with HTStream v.1.1.0 (https://s4hts.github.io/HTStream/ (accessed on 15 August 2019)) to perform sequence data QA/QC, remove adapter contamination and low-quality bases/sequences. The trimmed reads were aligned to the Vitis vinifera 12X genome (GCA_000003745.2, with Ensembl v.42 annotation, downloaded from https://plants.ensembl.org/Vitis_vinifera (accessed on 15 August 2019)) using STAR v.2.6.1d [71], to generate raw counts of reads per gene for each sample.

Differential expression (DE) analysis was conducted using the package limma-voom in R. Significant (*p* < 0.01) DEGs were analyzed for gene ontology using the PANTHER website [72,73]. The conserved responses to GRBV were determined by creating a Venn Diagram [74]. The WGCNA analysis was conducted in R using log2 counts per million reads and included all genes in the DE analyses. The analysis used a signed network and a robust biweight midcorrelation. A soft-thresholding power of 32 was chosen, using the WGCNA function pickSoftThreshold, as the smallest power for which the scale-free topology index exceeded 0.85. Differential co-expression analyses were conducted in R using the z-score method [75] as implemented in the Bioconductor package dcanr [76], version 1.6.0, which compares correlation coefficients between pairs of genes across conditions. *p*-values were adjusted for multiple testing using the Benjamini-Hochberg method [77].

Subsequently, the reads that did not align to the grape genome were aligned to the GRBV RefSeq genome sequence (https://www.ncbi.nlm.nih.gov/data-hub/genome/GCF_000909815.1/ (accessed on 18 November 2019)) using Bowtie2 v.2.3.4.1 [78]. The featureCounts tool of Subread v.1.6.3 [79] was used to assign the read alignments to open reading frames and to generate raw viral counts, which were used as input to the statistical analysis.

## 5. Conclusions

GRBV is the first geminivirus detected in grapevines, and our understanding of the detrimental impacts of the virus on grape and wine composition and quality is advancing. In this study, the seasonal impact was larger than the genotypic impact of rootstock on GRBD induced changes in grapes. Seasonal differences considerably impacted disease outcomes in grapevines, where the impact in 2016 was larger than in 2017. Fewer differences in primary metabolites and the grape transcriptome between RB(+) grapes and RB(−) grapes in 2017 were concurrent with increased induction of a gene that encodes for DCL-2. CS on 420A rootstock was less sensitive to GRBV infection than CS on 110R rootstock, specifically in 2017. This was seen in anthocyanin accumulation and the grape transcriptome, specifically with plant-pathogen interactions in 2017. We hypothesize that the difference in vigor and drought resistance in the two rootstocks led to a difference in the microclimate of the grapevine and berry metabolism.

In past research, decreases in symptoms have been correlated to upregulation of RNA silencing. Our research potentially indicates the same phenomenon in grapevines infected with GRBV, where higher temperatures potentially led to induction of virus-induced RNA gene silencing in GBRV infected fruit and fewer differences in the grape transcriptome. In the current study, the transcript that encodes DCL-2 was only significantly upregulated at veraison in both rootstocks and seasons, which resulted in decreases in viral gene expression, suggesting a potential phenological control over plant-derived immune responses. Further work on calcium-binding proteins, RNA-induced gene silencing, and siRNAs analysis in GRBV infected grapes is needed to confirm these findings and obtain a holistic view of the plant-pathogen interactions during GRBV infection.

## Figures and Tables

**Table 1:** Log fold change of VIT_04s0023g00920 which encodes for a dicer-like protein (DCL2). Bolded values indicate a significant difference (FDR adjusted *p* < 0.05). DEG = differential expression, 110R = Cabernet Sauvignon on rootstock 110R, 420A = Cabernet Sauvignon on rootstock 420A, PV = pre-veraison, V = veraison, PoV = post-veraison, and H = harvest.

**DE of VIT_04s0023g00920** DCL2	Pre-veraison				Veraison				Post-veraison		Harvest			
	110R		420A		110R		420A		110R	420A	110R		420A	
	2016	2017	2016	2017	2016	2017	2016	2017	2016	2016	2016	2017	2016	2017
	1.03	0.55	0.28	−0.33	**1.39**	**2.33**	**1.94**	**2.08**	0.44	0.78	−0.02	−0.53	−0.68	−1.09

## Supplementary Materials

The following supporting information can be downloaded at: https://www.mdpi.com/article/10.3390/ijms232113248/s1.
