# Supplementary material for: Investigating Grapevine Red Blotch Virus Infection in Vitis vinifera L. cv. Cabernet Sauvignon Grapes: A Multi-Omics Approach
Source: Int J Mol Sci. 2022 Oct 31;23(21):13248. doi: 10.3390/ijms232113248 (PMC9658657; doi:10.3390/ijms232113248)
Supplement: Supplementary file 1 [file ijms-23-13248-s001.zip › FigureS2.pdf]

Gene	Functional annotation	Pre-veraison				Veraison				Post-veraison		Harvest			
		110R		420A		110R		420A		110R	420A	110R		420A	
		2016	2017	2016	2017	2016	2017	2016	2017	2016		2016	2017	2016	2017
VIT_06s0004g02620	Phenylalanine lyase 1 (PAL1)	**1.89**	0.07	1.08	0.65	-0.28	-0.75	-0.44	**-1.29**	-0.17	-0.27	0.31	0.30	**1.48**	0.51
VIT_13s0019g04460	Phenylalanine lyase 7 (PAL7)	1.39	-0.12	-0.60	0.26	-0.46	-0.52	0.05	-1.09	-0.15	-0.19	-0.16	0.13	1.12	0.38
VIT_08s0040g01710	Phenylalanine lyase 2 (PAL2)	0.92	0.36	-0.53	0.10	-1.11	-0.85	-0.02	-0.76	-0.54	-0.45	-0.86	-0.86	-0.77	-0.46
VIT_11s0065g00350	*Trans*- cinnamate 4-monooxygenase (C4H)	0.49	0.88	0.39	0.47	-1.99	-0.23	0.15	-1.04	-0.34	-0.23	-0.22	-0.44	-0.25	-0.61
VIT_02s0025g02920	Caffeic acid 3-O-methyltransferase (COMT)	-1.09	-0.33	-0.11	0.48	-0.74	-0.18	-0.05	-0.66	0.11	0.18	-0.69	-0.02	-0.25	-0.21
VIT_16s0098g00850	Caffeic acid 3-O-methyltransferase (COMT)	0.48	-0.19	0.00	-0.11	-0.31	-0.48	-0.48	0.33	0.28	0.35	**0.83**	0.52	0.36	0.92
VIT_04s0023g02900	Ferulate-5-hydroxylase (F5H)	-0.36	-0.42	0.11	-0.02	-1.94	0.78	0.78	-0.12	0.35	-0.51	-0.93	-1.09	-0.82	-0.35
VIT_06s0061g00450	4-Coumaroyl-CoA ligase (4CL)	0.39	-0.37	0.16	0.22	-0.07	0.53	0.23	**0.78**	0.15	0.37	0.55	0.32	0.24	0.23
VIT_11s0052g01090	4-Coumaroyl-CoA ligase (4CL)	0.69	0.18	-0.05	0.04	-0.93	-0.60	0.27	-1.15	-0.06	-0.49	-0.88	-0.70	-0.66	-0.09
VIT_12s0035g02070	Cinnamoyl-CoA reductase (CCR)	-0.56	-0.22	-0.02	0.48	0.09	0.30	0.44	-0.13	1.05	0.79	0.12	0.15	0.21	0.15
VIT_14s0066g01150	Cinnamoyl-CoA reductase (CCR)	0.36	0.85	0.89	0.43	**-1.76**	-0.97	0.87	-1.32	-0.83	-0.52	-0.63	0.11	-0.30	-0.96
VIT_02s0012g01570	Cinnamoyl-CoA reductase (CCR)	-0.43	-1.11	-0.42	0.45	-0.92	-0.73	-0.07	-0.73	0.17	0.10	-0.17	0.07	0.96	0.37
VIT_03s0180g00260	Cinamyl alcohol dehydrogenase (CAD)	-0.27	-0.01	-0.54	-0.13	-0.11	-0.22	0.16	-0.06	0.08	-0.18	0.04	0.19	-0.17	0.26
VIT_08s0040g00780	*P*- Coumaroyl shikimate 3'-hydroxylase isoform	0.74	0.52	-0.21	-0.21	**-2.02**	-0.75	0.50	-1.02	0.47	-0.16	0.40	-0.66	-0.35	-0.43
VIT_16s0100g01030	Stilbene synthase (STS)	-0.27	0.37	-0.22	1.55	-1.55	-0.64	-0.16	-1.93	1.14	-0.17	1.79	-0.84	1.19	0.23
VIT_16s0100g01200	Stilbene synthase (STS)	0.58	0.92	0.50	0.45	-0.93	-0.47	0.15	-1.56	0.16	-0.04	1.83	-1.67	-2.04	-0.71
VIT_14s0068g00930	Chalcone synthase 1 (CHS1)	1.80	0.60	1.48	-0.46	-1.54	-0.91	0.00	-0.95	-0.38	-0.31	0.45	-0.23	0.73	0.42
VIT_05s0136g00260	Chalcone synthase 3 (CHS3)	**1.19**	-0.46	0.60	-0.24	-0.32	-0.57	-0.05	-0.94	0.22	0.25	0.57	0.62	**1.51**	0.42
VIT_13s0067g03820	Chalcone isomerase (CHI)	0.39	0.17	-0.01	-0.25	-0.23	-0.52	0.11	-0.86	0.27	0.01	0.49	0.36	**0.93**	0.10
VIT_13s0067g02870	Chalcone isomerase 2 (CHI2)	**0.57**	0.38	0.23	-0.04	-0.17	-0.40	-0.03	**-0.65**	0.46	0.35	1.30	0.57	**2.00**	0.33
VIT_04s0023g03370	Flavanone 3-hydroxylase (F3H)	**0.95**	0.08	0.60	-0.19	-0.17	-0.10	-0.16	-0.98	-0.15	0.06	0.05	0.26	**1.01**	0.36
VIT_18s0001g12800	Dihydroflavanol 4-reductase (DFR)	**0.82**	-0.03	-0.04	-0.09	-0.18	0.02	-0.30	-0.22	0.03	0.21	-0.29	-0.32	**0.70**	-0.13
VIT_02s0025g04720	Anthocyanidin synthase (ANS)	**1.00**	-0.33	0.51	-0.09	-0.09	-0.45	0.04	-0.61	-0.01	0.02	0.59	0.39	**1.18**	0.46
VIT_00s0361g00040	Anthocyanidin reductase (ANR)	**1.38**	0.80	0.80	-0.12	0.21	-0.62	-0.47	-0.74	-0.02	0.54	0.12	0.34	-0.92	-0.91
VIT_16s0039g02230	UDP-glucose:anthocyanidin 3-O-D-glucosyltransferase (UF3GT)	0.74	-0.77	0.40	-0.30	-0.36	0.03	0.53	-0.99	0.25	0.03	0.63	0.82	1.22	0.10
VIT_12s0034g00130	UDP-glucose:anthocyanidin 3-O-D-glucosyltransferase (UF3GT)	-0.23	0.30	-0.55	0.88	**-1.81**	0.11	-0.49	-0.55	0.23	-0.30	-0.02	0.18	**-1.19**	-0.06
VIT_12s0055g00290	UDP-glucose:anthocyanidin 3-O-D-glucosyltransferase (UF3GT)	**-1.00**	-0.34	-0.52	-0.47	-0.03	0.25	-0.08	-0.99	-0.11	-0.76	0.09	-0.07	-0.04	-0.41
