# Supplementary figures and images for: Investigating Grapevine Red Blotch Virus Infection in Vitis vinifera L. cv. Cabernet Sauvignon Grapes: A Multi-Omics Approach
Source: Int J Mol Sci. 2022 Oct 31;23(21):13248. doi: 10.3390/ijms232113248 (PMC9658657; doi:10.3390/ijms232113248)

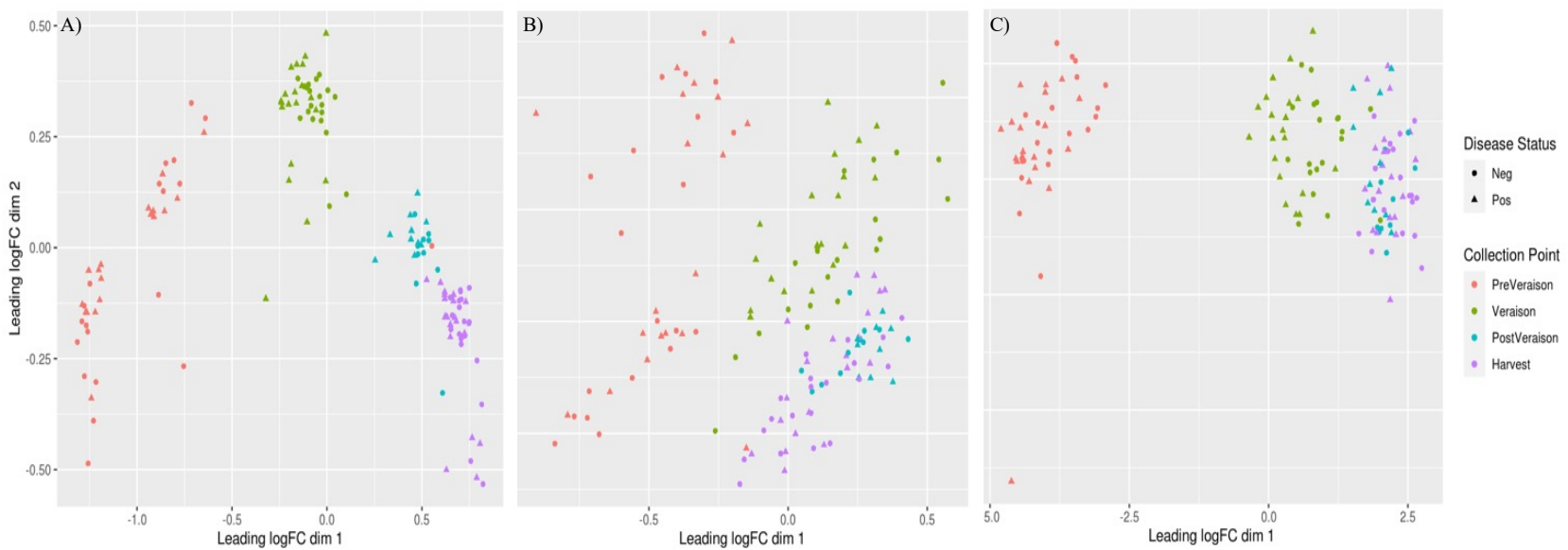

Supplement: Supplementary file 1 [file ijms-23-13248-s001.zip › FigureS1.pdf]

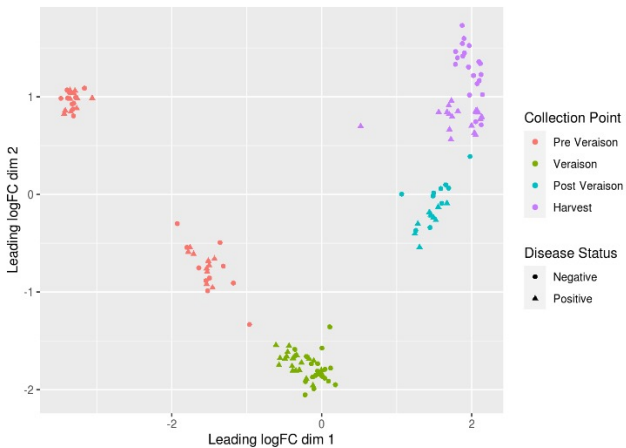

Supplement: Supplementary file 1 [file ijms-23-13248-s001.zip › FigureS3.pdf]

# Cluster Dendrogram

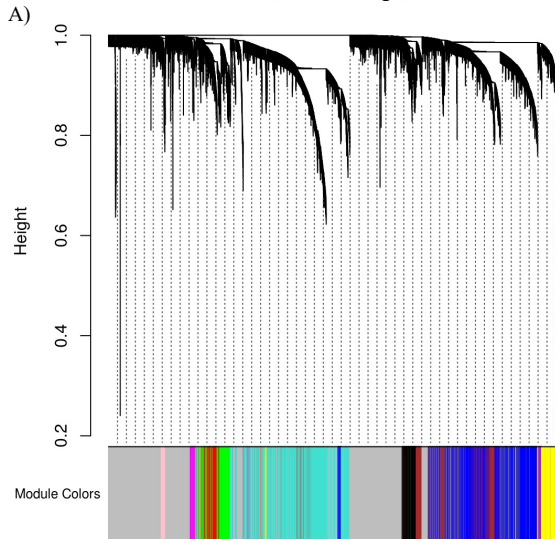

B)

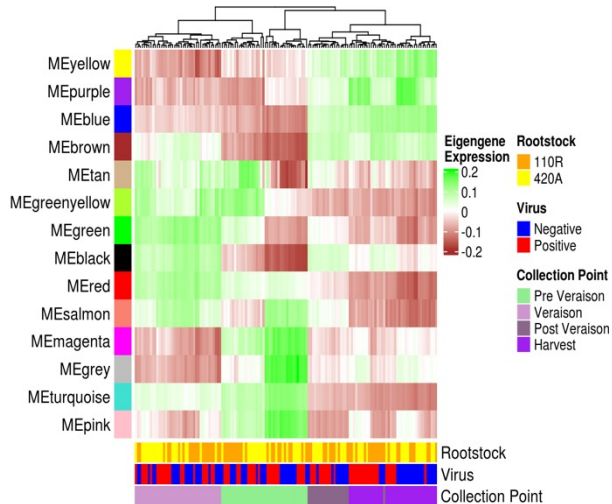

Supplement: Supplementary file 1 [file ijms-23-13248-s001.zip › FigureS4.pdf]

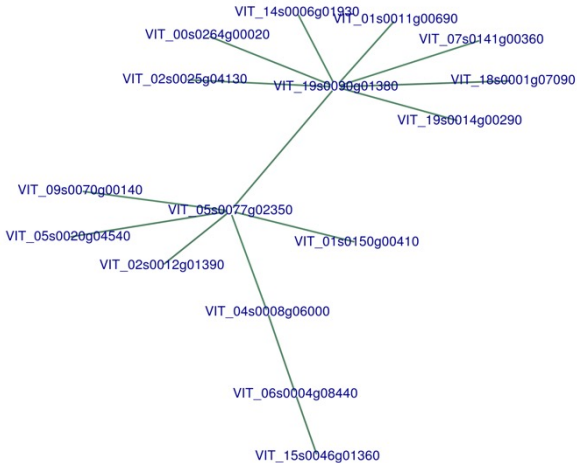

Supplement: Supplementary file 1 [file ijms-23-13248-s001.zip › FigureS5.pdf]

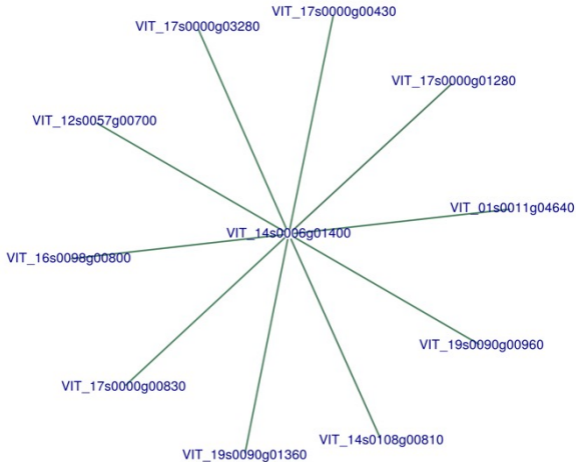

Supplement: Supplementary file 1 [file ijms-23-13248-s001.zip › FigureS6.pdf]
